# Aging-friendly cities: Investigating the effects of street usage on the psychological satisfaction of older adults in megacities

**DOI:** 10.3389/fpsyg.2022.942301

**Published:** 2022-09-20

**Authors:** Jian Li, Xing Gao, Yue Qiu, Yantao Ling

**Affiliations:** ^1^School of Humanities and Social Sciences, Beijing Institute of Technology, Beijing, China; ^2^Beijing Institute of Technology, Beijing, China; ^3^School of Management and Economics, Beijing Institute of Technology, Beijing, China; ^4^School of English Language, Literature and Culture, Beijing International Studies University, Beijing, China; ^5^School of Economics and Finance, Chongqing University of Technology, Chongqing, China

**Keywords:** psychological satisfaction, street usage, older adults, street type, megacities

## Abstract

The psychological satisfaction of older adults is an important evaluation standard for the construction of elder-friendly cities. Meanwhile, as important space carriers carrying the travel activities and social participation of older adults, streets are also of great significance to improve psychological wellbeing. However, few studies pay attention to the street usage of aging population, especially in the context of megacities. Moreover, the previous literature rarely distinguishes the types of streets. Thus, employing a mixed approach, we investigate the relationships between street usage and psychological satisfaction for older adults. Based on the survey in Shanghai, we find that a clear role for different usage indicators in the determination of subjective psychological satisfaction of older adults. More specially, the street usage and psychological satisfaction for older adults are strongly correlated, especially for living streets. Psychological satisfaction for older adults in different types of streets is not always positively related to the positive perception of street usage. The psychological satisfaction of different streets depends on different factors. By focusing on the case of a megacity, our study emphasizes the differences between different types of streets, which will be conducive to the proposal of practical planning policies. In addition, employing mixed research methods not only explains how different street usage affects the psychological welfare of older adults on a macro scale, but also emphasizes the inner world of respondents.

## Introduction

The wellbeing of aging people is an important dimension of modern city development and planning in response to increasing urbanization and the aging population (Kemperman and Timmermans, 2014; Alidoust et al., 2019). Psychological satisfaction with aspects of urban life (e.g., street usage) is an effective measure of a society’s ability to promote wellbeing and harmony. Psychological satisfaction of older adults is also widely regarded as a predictor of social inclusion because psychological satisfaction can be enhanced by increasing the mobility and independence of vulnerable groups within the built environment (Chen et al., 2019; Zhang et al., 2022). Thus, many studies have explored the wellbeing of older adults in the development process of cities and regions from the perspectives of community support and health services (Sexton and Bedford, 2016), family pressure (Santis, 2012), voluntary or paid work (Sagherian et al., 2021), and consumption and economy (Delabastita et al., 2021). Studies have all acknowledged that progress in the built environment correlates with improved psychological wellbeing for older adults.

Population aging has become a global social phenomenon in modern urban development. Results of China’s seventh census in 2020 indicated that the proportion of older adults in the population had reached 13.5%, with an increase of 4.63% from the previous census. Growth of China’s aging population is accelerating. Shanghai is a megacity with the largest proportion of older adults in China. By the end of 2020, there were 53,349 million elderly people (aged 60 and above), accounting for 36.1% of the total residential population. The number of elderly people living alone was 305,200 (Zhu et al., 2022). It is estimated that by 2035 and 2050, China’s elderly population (aged 65 and over) will reach 327 million and 393 million, respectively, accounting for 21.8 and 26.2% of the global elderly population (Ren, 2022).

An aging population has more intensive and frequent street usage interactions. However, energy and physical constraints mean older people face a higher risk of social exclusion in their street usage that can result in physical and psychological problems, ultimately reducing their quality of life. Consequently, improving the quality of street design and infrastructure for older adults has become an important policy goal of urban planning. The World Health Organization (WHO) (2002) also advocated for access to health security for the world’s aging population, including people in rural areas. In a society with an increasing proportion of older adults, there is an urgent demand for improved planning for this changing demographic and a need for increased attention to the psychological health of the aging population.

Walking accounts for the greatest absolute proportion of travel modes used by older adults (Feng, 2017). Therefore, older people have more time to come into contact with, use and experience the streets. Psychological satisfaction with street usage has been discussed in terms of three aspects—green space on the street, the improvement of street facilities and the construction of street atmosphere. From a green space perspective, trees and plants are considered a positive urban asset that can reduce negative psychological conditions (Taylor et al., 2015). Moreover, higher public green space cleanliness is associated with a lower incidence of depression (Afrad and Kawazoe, 2020). Lack of high-quality street facilities and environments may also lead to anxiety and depression, resulting in low-quality subjective perception and social psychological problems (Ellaway et al., 2009). Street atmosphere is affected by architectural environment and socio-economic status, and an appropriate street atmosphere can improve residents’ sense of local identity and belonging (Williams and Kitchen, 2012).

However, previous studies are limited in several ways. First, most studies were not undertaken in the context of megacities, which reflect rapid urbanization and the iteration of information technology. Street design and planning must be adapted to conform with these changes in contemporary society. Older adults may find it difficult to adapt to the information society. They frequently encounter obstacles in the process of street usage that can lead to feelings of loneliness and isolation. Therefore, the study of street usage and psychological satisfaction in megacities is also of great significance for small and medium-sized cities. Second, type of street was seldom indicated in previous studies. The design and use of streets vary, and older adults use different types of streets differently. It is difficult to develop practical street improvement proposals for increased psychological satisfaction of older adults if we do not distinguish between street types. Third, most studies have used quantitative statistical analysis methods. Although such approaches can reveal overall trends, they do not provide insight into individuals and previous work has not provided a deep understanding of the factors that affect individual psychological satisfaction.

Our study aimed to investigate the effects of street usage on the psychological satisfaction of older adults, using Shanghai as a case study. The wellbeing of older adults can be expressed in various forms. Our study focused on the subjective manifestation of wellbeing, that is, perceived psychological satisfaction. Based on our field work, including questionnaires and interviews, we first employed correlation analysis to clarify how street usage factors correlate with psychological satisfaction in older adults. Then, ordinary least squares (OLS) estimation was used to reveal to what extent and how different street usage factors affect the psychological satisfaction of older adults. Finally, interviews were used to focus on individual psychological satisfaction and its formation mechanism. Our study contributes to the existing knowledge in several ways. First, the megacity context may provide a valuable reference for other cities. Second, we emphasized the differences between street types, which will be conducive to the proposal of practical planning policies. Third, we implemented a mixed research strategy combining quantitative and qualitative methods. We considered that this research strategy would yield complementary insights and open the possibility of using this research paradigm in psychology and related disciplines in the future. The remainder of the paper is organized as follows. Section 2 provides a literature review. Our study area, data and methods are clarified in Section 3. Section 4 describes the results of statistical analysis and interviews, together with discussion of these aspects. Finally, we conclude by summarizing significant findings and making suggestions for future research.

## Literature review

### Aging-friendly cities and psychological satisfaction

**FIGURE 1:**
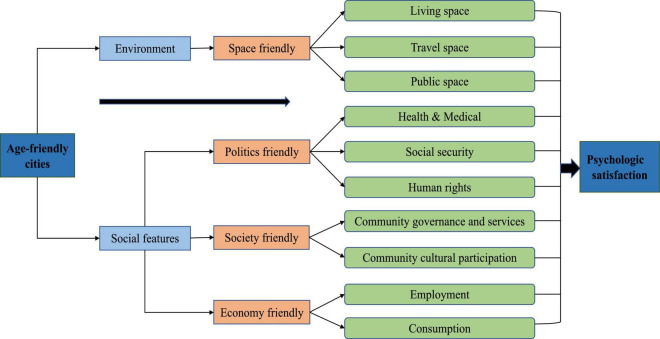
How do age-friendly cities lead to psychological satisfaction? (Source: Authors).

In this manuscript, we refer to “cities” as a general concept that includes traditional cities and urban communities. Aging is not a simple physiological reflection but rather a complex phenomenon constructed by social and historical environments (Wahl et al., 2012). As the economic environment has changed, so the social view of aging has gradually shifted from negative to positive. Older adults have slowly moved from “being regarded as a social burden” to “an important resource for families and society” (Yuan, 2021). Therefore, the construction of age-friendly cities is gradually becoming popular and occurring all over the world. Age-friendly cities aim to meet the needs of older adults, improve service quality, and create opportunities for older people to realize their self-worth. Such approaches promote active aging and enhance the psychological wellbeing and sense of acquisition of older adults (Buffel and Phillipson, 2016). These goals are brought about by optimizing the material environment, improving the policy system, and creating an atmosphere conducive to participation. Age-friendly cities include environmental and social features (Fitzgerald and Caro, 2014; Steels, 2015). Figure 1 illustrates how age-friendly cities lead to psychological satisfaction, and the main themes that formed the focus of previous studies.

Being “space friendly” is key in terms of environment. Studies of older peoples’ satisfaction with their living environment have developed from demographic, neighborhood, and social perspectives (Bruin and Cook, 1997; Phillips et al., 2005; Rioux, 2007). Living space is often the central focus of the aging population—especially people with physical and financial limitations—and thus, understanding how living space predicts psychological satisfaction is important (Rioux and Werner, 2011). Current studies are carried out at the level of state and ethnic groups, and travel space mainly focuses on how accessibility contributes to wellbeing. Accessibility is mainly viewed as the potential for opportunities to interact (Pereira, 2019), but it also involves land use, transport, and individual and temporal aspects (Geurs and Van Wee, 2004). The most basic logic is the extent to which the interaction between land use and transportation helps elderly individuals participate in social activities at different times to improve their psychological wellbeing (Zhang et al., 2022).

In terms of public space, systematic environmental construction can be used as a balanced means to address gender differences and improve psychological satisfaction in older residential communities (Yu et al., 2021). Public space promotes the health of older adults and determines their preferences through active aging approaches, especially their use of public space in residential areas (Lak et al., 2020). Cities can create a safe and accessible material space to improve the psychological wellbeing of older adults, taking the living place as the axis, the living community as the radius, and relying on urban public facilities. Planning that considers the wellbeing of older adults can ensure a comfortable, convenient, safe and worry-free urban environment for daily life and travel.

The study considered the links between age-friendly cities and psychological satisfaction from political, social and economic perspectives. The political perspective is mainly reflected by supportive and inclusive social environments. Studies aimed to improve psychological satisfaction through pension and medical services (Chen et al., 2020; Thomas et al., 2021) because the older population relies more heavily on health facilities than other population groups in urban construction (Hudson and Nolan, 2015). Further, social security involves public finance and government budget, including medical insurance, subsidies, and legal aid for older adults (Yang et al., 2010). Studies show that an optimal social security system can improve the psychological satisfaction of older adults by ensuring their life autonomy (Hsieh, 2015; Martin et al., 2021). Human rights imply a sense of respect for older adults, including access to basic public services in a range of places and through consideration of national public policies. This respect and ease of access can relieve the anxiety and psychological distress of older adults (Fernández-Fernández et al., 2020). The social-friendly perspective leads to the social participation of older adults in volunteer organizations, associations, and sports activities.

Social participation, including the comprehensive interaction between personal health and related environmental characteristics, plays an increasingly important role in promoting the psychological health and quality of life of older adults (Xin and Li, 2021). Moreover, health has a significant regulatory effect on the psychological and social compensation mechanism of older adults’ social participation (Xin and Li, 2021). In community culture, religious participation is important in alleviating the pain of older adults with low levels of social support, and religious participation may be important in managing the psychological distress of older adults (Dulin, 2005). Other studies have considered psychological satisfaction in the national cultures (Aw et al., 2017; Tsubouchi et al., 2021). To date, most studies have explored the relationship between consumption and psychological welfare of older adults, e.g., leisure, consumption of health products and living materials (Kekäläinen et al., 2016; Boehm et al., 2018). Few studies have investigated the reemployment of older adults, an important focus because it can increase older adults’ psychological satisfaction through the embodiment of their social value.

### Street space usage and psychological satisfaction

**FIGURE 2:**
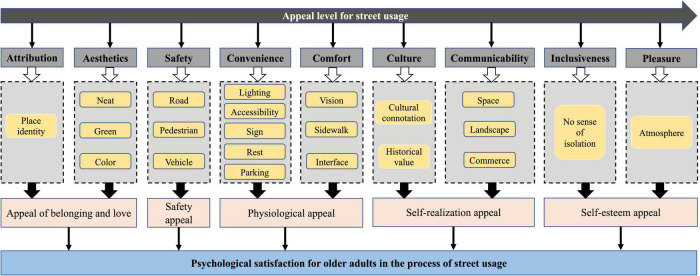
Street usage promoting psychological satisfaction for older adults (Source: Authors).

Based on research at city level, studies have focused on a smaller spatial scale: street usage (Tan, 2014; Carmona, 2019). Figure 2 demonstrates the relationships between street space usage and psychological satisfaction for older adults based on existing studies.

Streets represent the vitality of a city. A vibrant street should not provide a single traffic function but rather a combination of uses. Street space design should be people-oriented and pay attention to the needs of street users. Good street space design can enhance people’s travel and wellbeing. Studies of street usage have shifted from paying attention to a single demand for safety or belonging to more complex needs. For example, the safety and attractiveness of streets are important for increasing residents’ outdoor activities and perceived satisfaction (Rahm et al., 2020). In addition, improving the spatial planning of streets, such as spatial accessibility, green space, landscape configuration, and public facilities, can significantly improve the mental health of residents (Jeong et al., 2022; Kumar and Shukla, 2022; Zhang et al., 2022). Some studies have even discussed the relationship between street space use and high obesity rates (Zhao et al., 2018; Poulain et al., 2020).

Safety is the premise and foundation of all activities and is a key factor in determining older adults’ willingness to use the street. Many street safety theories and achievements relate to traffic vehicle and environmental safety, with traffic safety being a necessary condition for older adults to walk (Zhang et al., 2022). Safety involves the physical environment of the street, such as street materials and flat surfaces. Convenience mainly refers to the ease with which the street’s own facilities are used. Essentially, convenience leads to street users carrying out activities in the street and promotes the use and evaluation of street space (Landau, 2018). Physical and psychological comfort refers to the sense of comfort and tactile awareness of street use (Rodríguez Algeciras et al., 2016).

A good physical environment can provide users with a better street space experience. Street space provides the most basic transportation function in a city and is also the most important public activity space. It enables residents’ daily activities, such as social communication, and gives rise to primary impressions of a city. Dull streets may suggest that a city has little to offer, while vibrant streets remind people of the city’s vitality (Li et al., 2021). The vitality value of a street also reflects the street’s communicability (Xia et al., 2020). A lively street can promote older adults’ social interaction and help reduce loneliness. Esthetics is the psychological feeling brought about by street users’ perceptions of the visual level of the environment (natural environment and construction environment) (Southon et al., 2017). The natural environment includes street greening and planting, while the construction environment comprises street style, color, and cleanliness. Belonging in elder-friendly streets is a spiritual feeling regarding sense of identity, belonging, familiarity and the feeling of warmth and being “at home” (Smets and Sneep, 2017).

Older adults may take pleasure in elder-friendly streets that are attractive and appealing. Such streets contrast with a monotonous street atmosphere, yielding a positive subjective psychological perception of the street space and its diverse social activities (Kang et al., 2021). Culture emphasizes that the elements of street space can represent its historicity, story or unique regional characteristics (Ross, 2021) that can arouse the cultural identity and memory cognition of older adults. However, few studies have explored the psychological perception of street culture among older adults. Inclusiveness is a measure in a more general sense. It shows that street space design is conducive to the harmonious coexistence of groups of different ages, occupations, and educational levels. Inclusive street spaces are friendly and inclusive of the activities and exchanges of older adults, children, women, and other vulnerable social groups. In such spaces, older adults have a sense of identity and acceptance and are willing to socialize with others.

## Methodology

We examined how perceived characteristics of street usage influence psychological satisfaction of older adults across the streets of Shanghai. The theoretical framework that motivated the study was based on the multiple values of elder-friendly streets and the demands of older adults on streets. Although theoretical links between street planning and psychological satisfaction have been well-investigated, aging has not been taken into account, especially in metropolitan areas with extremely active economies. Thus, the study sought evidence of relationships between street usage and psychological satisfaction of older adults in the context of megacities.

### Case study

In light of the importance of case selection, a Chinese megacity was chosen to highlight street usage and psychological satisfaction issues in a context of rapid aging population growth. Megacities are important for several reasons. First, the cost of aging is gradually becoming unaffordable in megacities. Therefore, the choice of a megacity has practical significance for providing timely policy implications for policymakers and planners in megacities on how to refine the governance and planning regime to improve psychological satisfaction. Second, lessons and experiences from a megacity may be relevant for other growing cities although megacities are relatively unique regarding social development. Megacities are like other municipalities in terms of rapid urbanization and aging population expansion, with their governing and planning authorities and pro-growth initiatives. Furthermore, growing cities may face similar population control issues. The challenges faced by megacities in the past may confront other municipalities in future. Indeed, recent years have seen a substantial increase in aging society in other cities. Third, the large and diversified population and cultural structure of megacities are conducive to the establishment of sound infrastructure and public service rules, helping to form an age-friendly street usage and governance system. This complex process may provide a useful reference for other types of cities.

**FIGURE 3:**
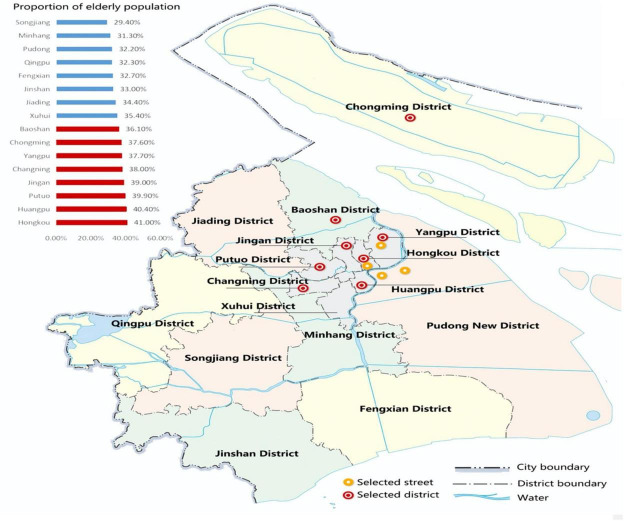
Study area and basic geographical and demographic information [Source: Zhu et al. (2022)].

Shanghai was chosen as our case study (Figure 3) because it provides a typical example of a world megacity that faces a serious aging issue (Zhu et al., 2022). Shanghai consists of 16 districts, and we selected four typical streets, all located in districts with a high proportion of older people. These streets were Rushan Road, Daxue Road, Hailun Road, and Century Avenue. Although older people may experience mobility difficulties, they have to adapt to the rapid socio-economic development of megacities. However, the psychological welfare of older adults in the built environment, especially their use of streets, has rarely been studied. While older adults play little role in the urban economy or construction, paying attention to their subjective welfare remains an important focus of urban planning and social psychology research. Furthermore, paying attention to the mental health of older adults in the process of environmental change will help build a full age-friendly city and maintain social stability. Such cities will also improve all residents’ sense of security.

The four streets selected in this study represent different street types, including living streets, cultural, creative and commercial streets, historical streets and landscaped streets. Rushan Road is a living street, surrounded by a large number of old communities built in the 1980s. It conveys a rich flavor of life with many real estate trading shops, fruit and vegetable shops, and other shops to meet daily needs. The shops are typically full of elderly people, many of whom are resting. In contrast, Daxue Road is a cultural, creative and commercial street. It is surrounded by residential housing, science and technology parks, and universities. There are many state-of-the-art modern public spaces on this street, but few elderly people stay or rest in these spaces. Hailun Road is a historical street built in 1908, with a rich history and culture. It is surrounded by residential communities and there are many elderly people on the street. Typically, older adults have specific street requirements. However, Hailun Road has few information guide signs; the sidewalk design lacks consideration of accessibility; there are few service facilities, such as rest seats, and there are no garbage cans on either side of the street. Finally, Century Avenue represents landscaped streets. It is a famous landscaped avenue built by the Shanghai municipal government and connecting the two landscaped areas of the Oriental Pearl TV Tower and the Century Park. It has public activity space for people to walk and enjoy their leisure or activities. The street was planned to maximize a comfortable walking experience. However, few elderly people linger on this street. Our study will distinguish the psychological satisfaction of older adults with these four street types.

### Data specification and variables

Our data were derived from a large-scale psychological satisfaction survey on street usage conducted by the authors. The design of our survey relied on the residential satisfaction survey implemented in Beijing in 2005, and the perceived psychological satisfaction with the prioritized information (Gao et al., 2021). Data collection was based on a questionnaire survey that took place in the four streets. The aim was to investigate older adults’ perceived psychological satisfaction with street usage. Considering the living habits of older adults and to increase the probability of obtaining an adequate sample, we only distributed the questionnaire on weekends, and at specific time periods, i.e., 7–10 a.m. and 2–5 p.m. To ensure balanced sampling, each street was assigned 120 questionnaires, and thus, we distributed a total of 480 questionnaires. To improve the validity of the questionnaire and the quality of data, we conducted a face-to-face questionnaire survey for each respondent. We were, thus, able to respond immediately and provide face-to-face explanations when elderly people had questions about the survey. The entire survey process lasted 4 months and we collected 471 valid questionnaires, with an effective rate of 98.1%.

This study mainly considered subjective perceptions and psychological evaluation of older adults on street usage. Variables were measured using subjective perception scores derived from the questionnaire based on the subjective evaluation of the respondents. To ensure a neutral evaluation point and make the descriptions more accurate, we selected an evaluation scale with 7 points, ranging from −3 to 3. The median value of 0 represents neutral perception. A positive value indicates positive perception, while a negative value indicates a negative view.

The dependent variable in the study was psychological satisfaction of older adults. The outcomes of psychological satisfaction were quantified by asking, “In the process of using the street, do you think the current design of the street satisfies you or makes you feel psychologically comfortable?” This type of measure of satisfaction is a vital tool for psychological growth and welfare in existing studies (Ryan and Deci, 2000; Zheng et al., 2022). In this study, psychological satisfaction refers to a measure of older adults’ overall satisfaction with street design.

Our independent variables comprised nine dimensions: attribution, esthetics, safety, convenience, comfort, culture, communicability, inclusiveness, and pleasure (Table 1). Attribution measured the psychological evaluation of older adults on their sense of belonging in the streets, ranging from “warmth” to “loneliness.” Esthetics included three aspects: environment, greening, and overall design. Environment refers to the psychological evaluation of street cleanliness ranging from “clean” to “dirty.” Greening is the perception and evaluation of street greening level, measured in terms of “more greening” to “less greening.” The overall design is a perceptual evaluation of the coordination of street features, ranging from “coordination” to “clutter.” Security consisted of four indicators. The first was the evaluation of road roughness, ranging from “flat” to “bumpy.” Second was the psychological evaluation of the possibility of accidental crime, ranging from “safety” to “danger.” Third was the evaluation of road safety, ranging from “convenient” to “inconvenient.” The last was the evaluation of the degree of street eye supervision over adverse events such as crime, ranging from “transparent” to “closed” vision.

**TABLE 1:** Indicator clarity of independent variables for street usage.

One-level indicator	Two-level indicator	Abbreviated code	Most positive perception (3)	Most negative perception (−3)
Attribution	Psychological evaluation of street belonging	PSB	Warmth	Loneliness
Esthetics	Psychological evaluation of street cleanliness	PSC	Clean	Dirty and messy
	Perception of street greening level	PSG	More greening	Less greening
	Perception of street style coordination	PSS	Harmonious style and color	Disordered style and color
Safety	Evaluation of pavement flatness	EPF	Flat	Bumpy
	Psychological perception of the possibility of accidents and crimes	PAC	Safety	Danger
	Evaluation of the convenience of crossing the road	PCR	Convenient crossing	Inconvenient crossing
	Evaluation of street eye’s supervision degree for adverse events such as crime	ESC	Visibility	Visual field closure
Convenience	Psychological perception of night lighting facilities	PNL	Bright	Dim
	Evaluation of barrier-free facilities	EBF	Smooth operator	Many obstacles
	Evaluation of street character guide signs and their guiding clarity	EGS	Clear	Vague
	Evaluation of the adequacy of street facilities for rest	ESF	Convenient to rest	Inconvenient to rest
Comfort	Perception of sidewalk occupancy	PSO	Spacious sidewalks	Narrow sidewalk
	Evaluation of streetlights during the day	ESL	Soft light	Dazzling light
	Perception of street interface continuity	PSI	Interface continuity	Interface dispersion
	Perception of street enclosure	PSE	Open	Closed
Culture	Perception of street culture and characteristics	PSC	Cultural characteristics	No cultural characteristics
Communicability	Perception of street stopping	PSH	Willing to stay	Pass in a hurry
	Evaluation of street vitality	ESV	Energetic	Desolate
	Evaluation of the degree of interaction between older adults on the street	EIE	Conversant	Indifferent
Inclusiveness	Evaluation of the inclusiveness of streets to older adults	EIS	Inclusion	Exclusion
Pleasure	Evaluation of the interest of streets	EIT	Interesting	Dull

Four indicators were selected to measure the perception of convenience of street use for older adults. First was the evaluation of night lighting facilities, which ranged from “bright” to “dark.” Second was the evaluation of the interference of obstacles with facilities, described from “unimpeded” to “full of obstacles.” Third was evaluation of the street text used in guide signs and its guiding clarity, ranging from “clear” to “fuzzy.” Fourth was evaluation of whether the facilities available for resting in the street are sufficient, ranging from “convenient” to “inconvenient” for resting.

Perception of street comfort also comprised four indicators. The first was the perception of sidewalk occupation, ranging from “spacious” to “narrow” sidewalks. The second was the evaluation of streetlights during the day, ranging from “soft” to “dazzling.” Third was the perception of street interface continuity, from “interface continuity” to “interface dispersion.” The fourth was perception of the sense of street enclosure, from “open” to “closed.” Culture refers to the perception of street culture and characteristics, ranging from “cultural” to “non-cultural” characteristics.

Communicability mainly measures the social attributes of streets and consists of three indicators. The first is the perception of street staying. The most positive evaluation was “willingness to stay,” while the most negative was “pass in a hurry.” The second is the evaluation of street vitality, which ranged from “vitality” to “desolation.” Finally, it evaluates the degree of interaction between older adults on the street. The most positive evaluation was “familiarity” while the most negative was “indifference.” Inclusiveness assesses how inclusive streets are for older adults, ranging from “inclusive” to “exclusive.” Lastly, the evaluation of street pleasure ranged from “interesting” to “boring.”

### Methods

This work lies at the intersection of social psychology and urban geography, and thus several methods were combined in keeping with the interdisciplinary approach. Quantitative methods included correlation analysis and Ordinary Least Squares (OLS) estimation, and qualitative methods involved semi-structured interviews. First, we computed the correlation coefficient for street usage and psychological satisfaction in older adults because psychological satisfaction can be regarded as a predictor of input in street usage and design. Thus, the degree of psychological satisfaction may directly influence street planning. Second, we employed OLS to explore the basic effects of street usage on psychological satisfaction of older adults. The OLS model specification was as follows:


(1) $$\mathrm{psychological}{\mathrm{satisfaction}}_{i}=\alpha +\sum_{k}\beta_{k}{\text{streetusage}}_{ki}+\varepsilon_{i}$$

Where, psychologicalsatisfaction_*i*_ is the psychological satisfaction for the older adult *i*; streetusage denotes a set of *k* independent variables, and the maximum value of *k* is 22. ε_*i*_ is the error term.

However, the results of OLS estimation are relatively weak in meeting our aim of testing the impact of street usage on psychological satisfaction because OLS regression may suffer from potential issues of biased estimation (Gao, 2021). Consequently, third, the study used semi-structured interviews to investigate how street usage affects the psychological satisfaction of older adults. The qualitative approach supplemented the quantitative methods, with qualitative interviews focusing on respondents’ views and exploration of events they consider relevant and important (Wang and Kulich, 2015). Researchers could flexibly adjust the interview questions, change the strategy and wording of questions, and even ask new questions (Clegg and Stevenson, 2013). Therefore, this approach helped us understand the study results more deeply.

## Results

### Correlation analysis

Table 2 shows the results of descriptive statistics concerning all variables. Table 3 indicates the results of correlation coefficients between street usage and psychological satisfaction. Table 2 indicates that street usage and the psychological satisfaction of older adults were strongly correlated, especially for living streets. Attribution was more strongly correlated with living and landscaped streets. Esthetics was more relevant to cultural, creative, and commercial streets and historical streets. In contrast, older adults have relatively low requirements for the safety of historical streets, possibly because of their low frequency of use of this type of street.

**TABLE 2:** Descriptive statistics.

Variables	Minimum	Maximum	Mean	Standard deviation
PSB	−3	3	0.954	1.7775
PSC	−3	3	1.44	1.571
PSG	−3	3	0.035	2.09
PSS	−3	3	0.473	1.835
EPF	−3	3	1.298	1.474
PAC	−3	3	0.421	1.978
PCR	−3	3	−0.367	1.926
ESC	−3	3	1.054	1.647
PNL	−3	3	1.263	1.851
EBF	−3	3	1.36	1.774
EGS	−3	3	0.15	1.868
ESF	−3	3	−1.723	1.928
PSO	−3	3	−0.075	2.316
ESL	−3	3	0.635	1.794
PSI	−3	3	0.575	2.056
PSE	−3	3	0.558	2.251
PSC	−3	3	0.773	2.074
PSH	−3	3	−0.148	2.591
ESV	−3	3	1.004	1.848
EIE	−3	3	0.642	1.943
EIS	−3	3	1.063	1.678
EIT	−3	3	0.583	1.975
Psychological satisfaction	−3	3	1.129	1.812

**TABLE 3:** Correlation coefficients for street usage and psychological satisfaction.

Variable	Psychological satisfaction of older adults			
	Living streets	Cultural, creative, and commercial streets	Historical streets	Landscaped streets
Attribution				
PSB	0.130**	0.062*	0.008**	0.085***
**Esthetics**				
PSC	−0.007	0.191**	−0.115	0.082**
PSG	0.143*	−0.159	0.075***	0.073
PSS	0.000	0.134**	−0.044	0.030*
**Safety**				
EPF	−0.140	0.052***	0.125*	0.112*
PAC	0.031***	0.046	−0.073	0.114*
PCR	0.144***	−0.015	0.033*	0.038*
ESC	−0.077	0.155**	0.047	0.092**
**Convenience**				
PNL	0.069**	−0.084	0.030**	0.194*
EBF	−0.003	0.054**	−0.045	0.102**
EGS	0.308***	−0.140	0.079**	0.109*
ESF	−0.123	0.045***	−0.081	−0.146
**Comfort**				
PSO	0.233*	0.190*	0.206*	0.190*
ESL	0.004**	−0.087	0.077**	−0.063
PSI	−0.173	0.009**	−0.008	0.112***
PSE	−0.120*	0.053	0.085	0.117*
**Culture**				
PSC	0.017*	0.012**	−0.071*	−0.058***
**Communicability**				
PSH	0.412**	−0.019	0.224*	0.031**
ESV	−0.022	−0.039	0.055	0.000
EIE	−0.065	0.020*	−0.072	0.046**
**Inclusiveness**				
EIS	0.039**	0.129	−0.030	0.007
**Pleasure**				
EIT	0.074***	0.159***	0.062**	0.052***

***Significant at 1% level, **5% level, and *10% level.

Increasing convenience can greatly improve the psychological satisfaction of older adults in living streets and cultural, creative and commercial streets, as indicated by the strong significance. Psychological satisfaction, particularly in living and landscaped streets, is positively related to comfort, whereas the other two streets have relatively low requirements for comfort. In terms of culture, the correlation results are interesting. Reduction in perception of street stopping (PSH) is significantly correlated with psychological satisfaction for older adults in historical and landscaped streets. One possible explanation is that high cultural connotation will increase the psychological burden of older adults and reduce their understanding of the streets in the process of street usage. Overall, although there is a significant correlation between communicability and psychological satisfaction, the degree of correlation was not strong. This may be because of the recent development and popularity of gated communities. In addition to living streets, inclusiveness was not significantly correlated with psychological satisfaction in other types of streets. In contrast, the increase in pleasure was significantly associated with psychological satisfaction of older adults in all types of streets.

### Ordinary least squares estimation results

Table 4 reports the OLS results on street usage impacting psychological satisfaction of older adults, based on street types. Contrary to our expectations, psychological satisfaction of older adults in different types of streets was not always positively related to the positive perception of street usage. Psychological evaluation of street belonging (PSB) was used to show attribution and had a positive effect on psychological satisfaction in all streets. The results indicate that positive attribution correlates significantly with high psychological satisfaction because a strong sense of belonging can bring feelings of familiarity and security, and lead to a relaxed mood (Harvey and Martinko, 2009; Burger et al., 2015). Esthetics had significant positive effects on living and landscaped streets, but its impact on cultural, creative and commercial streets, and historical streets was insignificant. In particular, the positive influence of PSB on living streets means that a clean-living environment can increase the psychological satisfaction of older adults.

**TABLE 4:** Ordinary Least Squares (OLS) results.

Variable	Psychological satisfaction of older adults			
	Living streets	Cultural, creative, and commercial streets	Historical streets	Landscaped streets
**Attribution**				
PSB	0.026**	0.057*	0.036**	0.035***
**Esthetics**				
PSC	0.073*	0.140	−0.186	0.131*
PSG	−0.088	−0.177	−0.465	0.032***
PSS	−0.007	0.066	−0.069	0.011
**Safety**				
EPF	−0.186	−0.083	0.180	0.151*
PAC	0.021**	0.050*	−0.234	0.071
PCR	0.217**	0.047*	0.023	−0.045
ESC	0.146***	−0.119	−0.105	0.037
**Convenience**				
PNL	0.104**	0.049	−0.042	−0.253
EBF	0.015*	−0.017	−0.222	0.600**
EGS	−0.237	0.043	0.227	−0.084
ESF	0.053***	0.094*	0.005**	0.107**
**Comfort**				
PSO	−0.339	0.129**	0.534	0.192*
ESL	−0.038	−0.158	0.034	0.004
PSI	−0.122	0.350	0.030	−0.092
PSE	0.179***	0.028	0.092	0.266**
**Culture**				
PSC	−0.067**	−0.057*	−0.081***	−0.045***
**Communicability**				
PSH	−0.637	−0.045	0.695	−0.018
ESV	−0.125	−0.086	0.078	0.015
EIE	0.091***	0.027	−0.115	0.031
**Inclusiveness**				
EIS	0.044**	0.141**	0.037***	0.023***
**Pleasure**				
EIT	−0.090	0.259*	0.111*	−0.051
R^2^	0.406	0.415	0.415	0.511

***Significant at 1% level, **5% level, and *10% level.

For landscaped streets, the positive effects of greening (perception of street greening level, PSG) were the most significant. These results have been widely discussed in literature that focuses on the relationships between green spaces and individuals’ psychological wellbeing (Qiao et al., 2021; Jiang and Huang, 2022). Living streets present significant demands for safety. In contrast, for older adults, the safety of historical streets has little to do with their psychological satisfaction. In cultural, creative and commercial streets, older adults are more concerned about accident crime and road safety because of the significance of psychological perception of the possibility of accidents and crimes (PAC) and evaluation of the convenience of crossing the road (PCR). In landscaped streets, flat pavements can significantly improve the psychological satisfaction of older adults. The convenience indicator of whether streets provide enough rest facilities can lead to significant change in older adults’ psychological satisfaction, and is determined by their physical function. In addition, in living streets, the improvement of night lighting facilities and fewer road obstacles can significantly increase older adults’ psychological satisfaction. Some variables in commercial streets were not significantly impacted because older adults rarely participate in commercial street activities. A similar situation exists on historical streets. Participants noted that even if they got lost, they would not look for signs but rather ask the people around them for help. Older adults reported rarely choosing to travel at night and, thus, the variable of psychological perception of night lighting facilities (PNL) was not significant in any street type.

At comfort level, the reality of tight land use in megacities means that open space and vision can lead to high psychological satisfaction in living streets. In cultural, creative and commercial streets, spacious sidewalks have a significant positive impact on the psychological satisfaction of older adults. High comfort does not necessarily increase psychological satisfaction with historical streets. To some extent, this reflects the limited interaction between older adults and historical streets. For landscaped streets, spacious sidewalks and wide horizons can increase psychological satisfaction. Culture has significant negative effects on the psychological satisfaction of older adults in all kinds of streets. Culture is not a pressing need for older adults using the streets (Hofer et al., 2016) and even a high cultural connotation will hinder older adults’ understanding and use of streets. Therefore, this negative impact suggests that street planners should consider the public’s acceptance of culture.

Living streets demand communicability, which has a significant positive impact on the psychological satisfaction of older adults. This result shows that having more familiar people on the streets leads to greater psychological satisfaction when using the streets. However, this significant impact only occurs in living streets. For other types of streets, communicability has no significant impact on psychological satisfaction. One possible reason is that older adults use these streets for a short time and seldom undertake social activities on these streets (Zhang et al., 2021). High inclusiveness levels were linked to high psychological satisfaction. Many elderly people find it difficult to accept modern information technology in the context of rapid economic development and the progress of smart megacities. They may experience negative psychosocial impacts of isolation (Östlund et al., 2015). Thus, when their needs are taken into account in the design or planning of streets, their psychological satisfaction will increase. Pleasure in cultural, creative and commercial streets and historical streets can lead to increased psychological satisfaction, although the effects of pleasure in living and landscaped streets were not significant.

### Interviews: How does street usage affect the psychological satisfaction of older adults?

Using interviews, we sought to understand more deeply how the factors in the questionnaire affect the psychological satisfaction of older adults. Results of OLS regression indicated that the psychological satisfaction of older adults was quite different for the four types of streets and each street had different characteristics. Overall, the factors had a more significant impact on living and landscaped streets, and general psychological satisfaction with historical streets was relatively low. These findings are consistent with the principle and original intention of our study design: diversified street models and environments are conducive to the design of street space suitable for older adults, and improving the psychological welfare of this population may lead to more all-age friendly cities. Interview results are presented in Table 5, showing the factors influencing the psychological satisfaction of older adults based on the indicators in the questionnaire.

**TABLE 5:** Interview results for psychological satisfaction of older adults in four types of streets.

Indicators	Psychological satisfaction of older adults			
	Living streets	Cultural, creative, and commercial streets	Historical streets	Landscaped streets
Positive	Warm, convenient to cross the street, transparent, energetic, familiar, and inclusive	Bright lighting at night, cultural characteristics, pleasure, and continuous interface	Cultural characteristics	Clean, green, flat, safe, clear vision, bright lighting at night, unobstructed, convenient for rest, clear signs, spacious and open sidewalks, willing to stay
Negative	Less greening, inconvenient rest, discrete interface, narrow sidewalk, and hurried passing	Inconvenient to rest, chaotic style and color, exclusion	Less greening, dangerous, inconvenient to cross the street, inconvenient to rest, dim lighting at night, blurred signs, narrow sidewalks, closed, hurried, cold, and boring	Inconvenient to cross the street and indifferent

**FIGURE 4:**
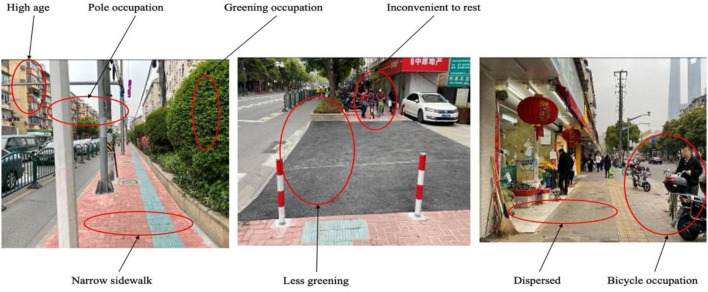
Street view of Rushan Road (Source: Authors).

Rushan Road is a living street in an old community in Shanghai where houses were built many decades ago. Most of the commercial activities on both sides of the street are closely related to residents’ daily lives, and most of the street users are older adults living in the community. Evaluation of the street included: (1) It is convenient to cross the street; (2) The line of sight is clear and the street is energetic; (3) Older adults are relatively familiar with shopkeepers and street pedestrians; and (4) The street is inclusive of older adults. However, there are also many deficiencies, mainly linked to the relatively old street construction. These included narrow sidewalks and road occupation by bicycles, telegraph poles and greening (Figure 4). Older adults expressed their dissatisfaction with aspects such as limited greening, few convenient resting places; discrete nature of the greening and pavement design, and inconvenient for walking. It is almost impossible to walk on the sidewalk if a person in a wheelchair is already using it. Other pedestrians then have to walk on the roadway, deepening insecurities about walking. Therefore, older adults are unwilling to stay on this street and limit their interactions to buying necessities and walking only when necessary in the area. On the whole, psychological satisfaction with Rushan Road is relatively low.

**FIGURE 5:**
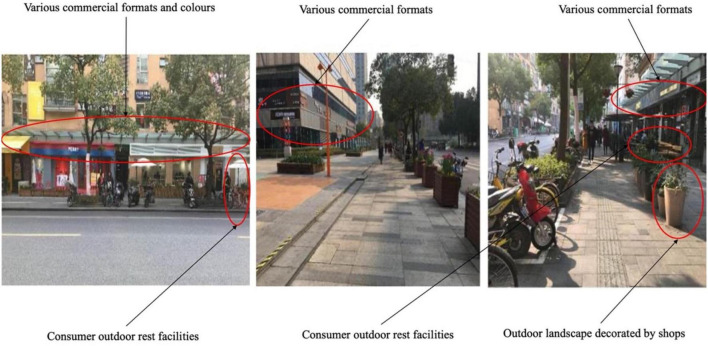
Street view of Daxue Road (Source: Authors).

Daxue Road is a cultural, creative and commercial street near Fudan University and Shanghai University of Finance and Economics. Both sides of this student street exhibit cultural and creative office land and residential buildings. Although most of the street users are young people, many elderly people walk among them. As shown in Figure 5, the businesses on both sides of the street are diverse, and many of the older people interviewed on this street noted that the lights are bright at night, and the commercial formats and store design have specific cultural characteristics that give them great pleasure. Shops are painted in different colors to show their personality, creating an impression of chaotic street style and color. Although the street is equipped with many rest and landscape facilities, most of them are part of shops and mainly for customers. Thus, older adults considered these facilities inconvenient places to rest and felt they were not generally inclusive.

**FIGURE 6:**
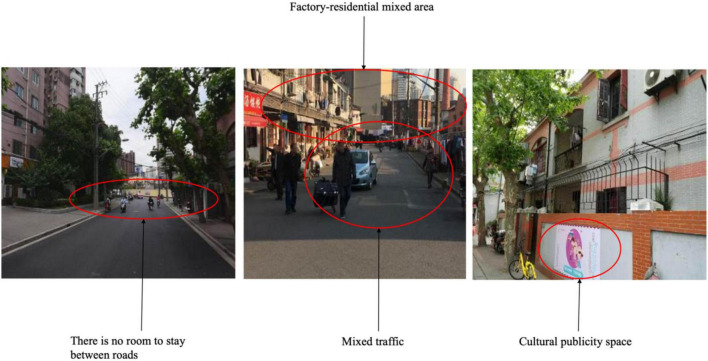
Street view of Hailun Road (Source: Authors).

In terms of historical streets, Hailun Road has a long history and rich culture. There are traditional mixed areas of factories and houses along the street and a former residence of calligrapher Shen Yinmo. The older adults interviewed on this section of the street referenced the area’s rich cultural characteristics. However, the streets have not been widened and the sidewalks are narrow with little greening. Pedestrians must sometimes walk in the carriageway—a situation even more inconvenient for older adults with mobility problems who need to use wheelchairs. The mixed traffic of people and vehicles on the high street complicated older adults’ psychological evaluation in terms of risk index and unsafe road crossing. Participants commented that the street is inconvenient for resting and there is little room to stop and chat (Figure 6). They must pass in a hurry in the street. Therefore, their perception of this street is relatively cold and boring, and the street has little attraction for older adults.

**FIGURE 7:**
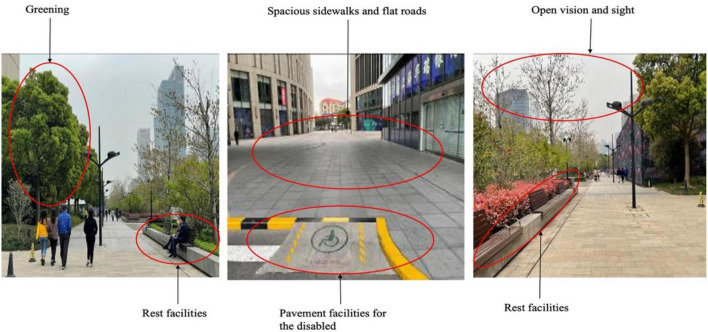
Street view of Century Avenue (Source: Authors).

Century Avenue is a landscaped urban avenue. Older adults were more positive about their psychological satisfaction of this space. Perceived impressions included open vision, clean streets, more greening, flat roads, transparent lines of sight, barrier-free facilities, clear street signs, unobstructed roads, convenient resting facilities, spacious sidewalks, clear division of people and vehicles, and bright night lighting (Figure 7). Walking on this street, they have a strong sense of security. The interviewees said they were willing to stay, talk and rest on the street. However, the landscaped avenue was also rated relatively low in overall psychological satisfaction. The wide street is difficult to cross and familiarity between pedestrians on the street is relatively low, leading to a limited sense of belonging and individuals not feeling at home on the street.

## Conclusion

The study aimed to evaluate the empirical evidence of street usage to account for older adults’ psychological satisfaction with different types of streets in Shanghai, China. Overall, our results demonstrate a clear role for usage indicators in determining subjective psychological satisfaction of older adults. Specifically, our study was conducted in the context of a megacity with rapid urbanization and a move to becoming a smart city. Knowing how older adults adapt to this intelligent society and street life is important for understanding their psychological satisfaction. Street segmentation was also an important contribution of this study. Using different types of streets, we explored the impact of street usage on psychological satisfaction, providing more detailed and feasible policy suggestions for the planning of streets. The mixed research methods explained how street usage affects the psychological wellbeing of older adults on a macro scale, and also enabled deep exploration of respondents’ inner worlds. In particular, the qualitative interviews showed the important role of interpretivism in psychological research.

Our study led to several conclusions. First, street usage and psychological satisfaction of older adults are strongly correlated, especially for living streets. This is because older adults use living streets the most, and thus, the intersection between older adults and living streets is great. Second, psychological satisfaction of older adults in different types of streets is not always positively related to their positive perception of street usage. In particular, culture was negatively correlated with older adults’ psychological satisfaction in historical and landscaped streets. This may be because older adults cannot fully understand the connotation of street culture in the process of street usage, leading them to feel isolated from society and increasing the risk of anxiety and depression. Third, the interview results indicated that in living streets, attribution, safety and inclusiveness lead to high psychological satisfaction, while esthetics, convenience, comfort, sociability, low pleasure, and culture lead to reduced psychological satisfaction. In terms of cultural, creative and commercial streets, pleasure and culture can bring high psychological satisfaction, whereas convenience, esthetics, and inclusiveness lead to low psychological satisfaction. In historical streets, increased culture improves the psychological satisfaction of older adults, while low safety, esthetics, convenience, comfort, communicability, and interest reduce psychological satisfaction. For landscaped streets, high esthetics, comfort, safety, and sociability have a positive impact on the psychological satisfaction of older adults, while low convenience leads to low satisfaction. Our findings suggest a need for a planning regime that pays more attention to the psychological satisfaction of older adults in the process of street usage. Table 6 summarizes the main study findings and their implications for city planning.

**TABLE 6:** Findings and planning implications.

Planning areas	Findings to consider for improving psychological satisfaction	Measures	Relevant government agencies
Space	• Traffic • Interface	• Sorting out the basic road system • Increasing the accessibility of street lines at different levels • Solving the occupation of street traffic space • Road zoning • The junction of street roads • The influence of street height-width ratio on elderly peoples’ perception of safety and comfort • Transparency and continuity of frontage interface	• Urban Transportation Department • Urban housing and Urban Rural Development Bureau
Policy	• Design orientation of streets • Governance policies	• Enhancing public and community participation of elderly people • Public space governance of communities and streets	• Neighborhood Committee • Subdistrict Office
Society	• Social communication • Infrastructure • Surrounding environment of the street	• Increasing the rest facilities on the street • Safe and comfortable sidewalk • Adequate and healthy lighting • Clear identification facilities • Barrier-free facilities • Greening facilities • Community health care and life circle planning	• Urban Transportation Department • Urban housing and Urban Rural Development Bureau • Municipal planning, education and public health departments
Economy	• Mobile vendor • Street business	• Increasing some elderly-oriented business projects • Space and time planning and management of mobile vendors	• Both municipal and relevant district planning, tax departments and DRC (development and reform commissions) • Municipal planning authority

Our results have other potentially important policy implications in improving the psychological satisfaction of older adults in street usage, especially in megacities. Traffic space, communication space, street facilities, street interface, and street surrounding functions require attention to address the psychological perceptions of older adults. Traffic space includes coherent and easy traffic lines, safe road zoning and barrier-free intersection design. The communication space mainly considers rest facilities with reasonable layouts, sidewalks with high trafficability and attractive street parks and squares. Street facilities mainly focus on bright lighting, clear identification, full coverage barrier-free facilities, convenient bus stops, and rich greening facilities. Street or frontage interface mainly includes pleasant interfaces with optimal height-width ratios, high continuity, and transparency. Finally, the diversity of street peripheral functions is also important for improving older adults’ psychological satisfaction.

Our study gives rise to suggestions for future research. For psychology, this research may promote intergenerational mobility and dialogue between age groups. Future research could include different age groups, using the same research design. More importantly, this study inspired us to include geographic information and urban planning in the research scope of psychological assessment. This was an exploratory expansion of the current research scope of social psychology. Quantitative methods can only provide a basic understanding of the relationship between street usage and psychological satisfaction, although endogenous issues specific to individuals may exist. Spatial autocorrelation and path dependence may have an important impact on the results because the current study was based on different geographical spaces. Future studies could solve the problem of endogeneity through instrumental variables, and spatial autocorrelation could be considered by introducing spatial weights.

## Data availability statement

The datasets presented in this article are available upon request. Requests to access the datasets should be directed to XG, xing.gao@bit.edu.cn.

## Author contributions

JL supervised the whole study. XG wrote the study. YQ hold data collection and revised the study. YL conducted data analysis. All authors contributed to the article and approved the submitted version.
